# Comparative Lipidomics Unveils Species-Specific Lipid Signatures in Three *Zanthoxylum* Species

**DOI:** 10.3390/foods15020372

**Published:** 2026-01-20

**Authors:** Guangbo Xie, Sijia Xie, Leilei Du, Chu Chen

**Affiliations:** 1 School of Life Science and Technology, University of Electronic Science and Technology of China, Chengdu 610054, China; a994318971@163.com; 2 School of Ethnic Medicine, Chengdu University of Traditional Chinese Medicine, Chengdu 611137, China; duleilei@cdutcm.edu.cn; 3 Sichuan-Chongqing Joint Key Laboratory of Innovation of New Drugs of Traditional Chinese Medicine, Sichuan Academy of Chinese Medicine Sciences, Chengdu 610041, China

**Keywords:** huajiao, *Zanthoxylum bungeanum*, *Zanthoxylum schinifolium*, *Zanthoxylum armatum*, lipidomics, fatty acid

## Abstract

*Zanthoxylum* species, commonly known as Sichuan pepper, are valued as food ingredients for their unique aroma and pungency. However, a comprehensive understanding of their lipid composition, which may serve as both flavor precursors and nutritional components, remains limited. In this study, we performed a comparative lipidomic analysis of three economically important *Zanthoxylum* species (*Z. bungeanum*, *Z. schinifolium*, and *Z. armatum*) using ultra-high-performance liquid chromatography coupled with high-resolution mass spectrometry. Fatty acids were concurrently analyzed by gas chromatography. A total of 315 lipid molecules were identified and categorized into 53 fatty acyls, 132 glycerolipids, 50 glycerophospholipids, 46 sphingolipids, and 34 sterol lipids. Triacylglycerols (22.84–54.25%) and free fatty acids (28.07–39.61%) were the most abundant lipid subclasses. Multivariate statistical analysis revealed 44 significantly different lipid molecules among the species, and pathway enrichment analysis indicated glycerolipid metabolism as the most significantly altered pathway. Furthermore, fatty acid profiling showed a nutritionally balanced n-6/n-3 polyunsaturated fatty acid ratio (1.04–1.12). These species-specific lipid signatures not only provide a basis for varietal authentication but also highlight the potential of *Zanthoxylum* lipids in shaping flavor profiles and contributing to nutritional value, supporting their diversified application in food products.

## 1. Introduction

Huajiao, commonly known as Sichuan pepper, refers to the oil-rich pericarp of certain *Zanthoxylum* species (Rutaceae). With a history of over 2000 years in Chinese culinary and medicinal tradition [1,2], it is highly valued for its characteristic sensory properties and bioactivities. Over recent decades, *Zanthoxylum* (Sichuan pepper) has become a major agricultural product in China. In 2022, the national production reached 574,000 tons, with a total value exceeding CNY 40 billion, and Sichuan Province led in both cultivation area and yield [3]. This substantial production is predominantly channeled into the food sector. Beyond direct use as a spice, it is extensively processed into forms such as Sichuan pepper oil, compound seasoning pastes, and hotpot bases, where its content is significant [4,5]. Consequently, a systematic characterization of its lipid profile—the key to flavor formation and potential nutritional value—is crucial for product quality, authenticity, and the development of lipid-based functional ingredients. Beyond its culinary significance, *Zanthoxylum* also holds official medicinal status in China. The dried ripe pericarps of both *Z. schinifolium* and *Z. bungeanum* are officially documented in the Chinese Pharmacopoeia for treating conditions such as stomachache and pruritus [6].

Red huajiao (RHJ, *Z. bungeanum*), green huajiao (GHJ, *Z. schinifolium*), and tengjiao (TJ, *Z. armatum*) are the most widely cultivated and consumed *Zanthoxylum* species in China. These three species exhibit distinct flavor profiles, influenced by their geographical distributions and morphological characteristics. RHJ, primarily cultivated in western and southwestern China, is characterized by an intense numbing sensation [7]. GHJ, mainly grown in southwestern regions, is distinguished by a fresh, pungent aroma and numbing taste [8]. TJ, extensively cultivated in southern China, possesses a unique sensory profile combining numbing effect with a fruity aroma [9].

Pharmacological studies have demonstrated that huajiao extracts possess various biological activities, including anti-inflammatory [10,11], antioxidant [12], antimicrobial [13], and anesthetic effects [14,15], largely attributed to their diverse secondary metabolites [2,16]. Previous phytochemical research on huajiao has primarily focused on terpenoids, alkaloids, coumarins, lignans, and flavonoids, aiming to elucidate the basis of its sensory properties and health benefits [1,2,16,17,18]. In contrast, lipids—fundamental as energy reserves and structural components of cellular membranes—have received comparatively little attention. Beyond basic metabolic functions, lipids also serve as signaling molecules and participate in plant communication [19]. Notably, lipids act as important flavor precursors; their oxidation and degradation generate volatile aroma compounds such as aldehydes and ketones, significantly contributing to huajiao’s unique fragrance and taste [20,21,22]. Furthermore, lipid composition can reflect ecological adaptability and quality variation among species, offering insights into the distinct characteristics of different huajiao varieties [23,24,25]. However, our current understanding of lipids in *Zanthoxylum* remains fragmented. While earlier gas chromatography studies indicated that dried huajiao pericarps and seeds are rich in unsaturated fatty acids like linoleic and oleic acid [26,27], a systematic and comprehensive lipidomic profile is still lacking. This creates a significant knowledge gap, disconnecting the extensive research on volatiles from the underlying lipid precursors that give rise to them.

Lipidomics, utilizing high-resolution mass spectrometry (e.g., UPLC-MS) coupled with multivariate statistics, enables efficient and precise identification and quantification of thousands of lipid species within complex biological matrices [28,29]. This approach has been successfully applied in food science to authenticate olive oil varieties, address process optimization issues in peanut oil processing, and trace flavor determinants in tea [30,31,32]. Compared to conventional methods, modern lipidomics allows for the simultaneous profiling of multiple lipid categories—including glycerolipids (GLs), glycerophospholipids (GPs), and sphingolipids (SPs)—and reveals dynamic metabolic networks, providing a powerful tool for elucidating lipid metabolic differentiation among huajiao species [23,33,34].

Building on advances in lipidomics [35], this study employed a UPLC-Q-TOF-MS/MS-based lipidomics approach to comprehensively characterize the lipid profiles in the fresh pericarps of three economically important *Zanthoxylum* species: red huajiao (RHJ, *Z. bungeanum*), green huajiao (GHJ, *Z. schinifolium*), and tengjiao (TJ, *Z. armatum*). The specific objectives were (1) to comprehensively characterize and compare their lipid profiles at the molecular level; (2) to identify lipid biomarkers that can discriminate among the three species; (3) to explore the key metabolic pathways underlying the observed lipid variations; and (4) to evaluate the fatty acid composition. Our work provides the first comparative lipidomic landscape of these species, addressing a critical knowledge gap and establishing a foundational dataset for future research on food authentication, nutritional assessment, quality control, and the development of functional lipid ingredients from *Zanthoxylum*.

## 2. Materials and Methods

### 2.1. Materials

Dichloromethane (CH_2_Cl_2_) and methanol (MeOH) of analytical grade were purchased from Merck (Rahway, NJ, USA). Acetonitrile (ACN), isopropanol (IPA), and ammonium acetate of LC-MS grade were obtained from Sigma-Aldrich (St. Louis, MO, USA). Ultrapure water was produced by a Mili-Q system (Bedford, MA, USA). Lipid internal standards, including fatty acid (FA d4 C16:0), triacylglycerol (TG 15:0/18:1(d7)/15:0), diacylglycerol (DG 15:0/18:1(d7)), monoacylglycerol (MG 18:1(d7)), phosphatidylcholine (PC 15:0/18:1(d7)), lysophosphatidylcholine (LPC 18:1(d7)), phosphatidylethanolamine (PE 15:0/18:1(d7)), lysophosphatidylethanolamine (LPE 18:1(d7)), phosphatidylinositol (PI 15:0/18:1(d7)), phosphatidylglycerol (PG 15:0/18:1(d7)), phosphatidylserine (PS 15:0/18:1(d7)), Cholesteryl Ester (18:1(d7)), C15 ceramide-d7, and sphingomyelin (SM d18:1/18:1(d9)) were purchased from Avanti Polar Lipids (Alabaster, AL, USA). A mixed methanol solution containing all lipid standards at 10 μg/mL each was prepared and stored at –20 °C.

Fresh fruits of three *Zanthoxylum* species—red huajiao (*Z. bungeanum*), green huajiao (*Z. schinifolium*), and tengjiao (*Z. armatum*)—were collected from their primary cultivation bases in Sichuan Province, during the peak maturation period (July 30—1 August 2024). Specifically, *Z. bungeanum* was sampled from Jiuxiang, Hanyuan County, Ya’an (29°33′ N, 102°37′E; elevation: 1475 m); *Z. schinifolium* from Gaoban, Jintang County, Chengdu (30°63′ N, 104°63′ E; elevation: 423 m); and *Z. armatum* from Zhongbao, Hongya County, Meishan (29°52′ N, 103°16′ E; elevation: 508 m) (Figure S1). All sampling sites feature distinct microclimatic and edaphic conditions conducive to *Zanthoxylum* cultivation. For each species, three biological replicates were collected. Each replicate comprised approximately 30 fresh fruits randomly harvested from multiple healthy plants within the standardized plantation. The samples were identified by Professor Chu Chen, a plant taxonomist at Sichuan Academy of Chinese Medicine Sciences, and stored at –80 °C prior to analysis.

### 2.2. Total Lipid Extraction

Total lipids were extracted from huajiao pericarps using a modified Folch method [36,37]. Briefly, seeds were removed from fresh fruits on ice, and the pericarps were ground into powder under liquid nitrogen. An aliquot of 50 mg powder was weighed into a glass tube, followed by the addition of an 80 μL (10 μg/mL) internal standard mixture, 2 mL of CH_2_Cl_2_, and 2 mL of MeOH. The mixture was vortexed for 1 h, after which 2 mL of CH_2_Cl_2_ and 1.6 mL of ultrapure water were added. After further vortexing and centrifugation at 5000 rpm for 10 min, the subnatant was collected and evaporated to dryness under a nitrogen stream. The residue was redissolved in 800 μL of CH_2_Cl_2_/MeOH (1:1, *v*/*v*) and filtered through a 0.22 μm syringe filter. All extractions were performed in triplicate as biological replicates (i.e., using separate fruits from different individual plants), and the extracts were stored at –20 °C until UPLC-Q-TOF-MS analysis.

### 2.3. UPLC-Q-TOF-MS Analysis for Lipid Profiling

Lipid separation was performed on a Shimadzu UPLC-30A system (Kyoto, Japan) equipped with a Kinetex C18 column (100 × 2.1 mm, 2.6 μm, Phenomenex, Torrance, CA, USA) and a corresponding Security Guard precolumn. The mobile phases consisted of (A) H_2_O/MeOH/ACN (1:1:1, *v*/*v*/*v*) containing 5 mM ammonium acetate and (B) IPA/ACN (5:1, *v*/*v*) containing 5 mM ammonium acetate. The gradient elution program was as follows: 0–0.5 min, 80% A; 0.5–1.5 min, 60% A; 1.5–2 min, 40% A; 2–10 min, 5% A; 10–12 min, 5% A; 12–13.1 min, 80% A, 13.1–14 min, 80% A. The flow rate was 0.4 mL/min, the autosampler temperature was maintained at 4 °C, and the column temperature was set at 60 °C. The injection volume was 3 μL.

Mass spectrometry analysis was conducted using a TripleTOF 6600 system (AB Sciex, Concord, ON, Canada) with an electrospray ionization (ESI) source operating in both positive and negative modes. The MS parameters were set as follows: ion spray voltage, +5500 V/−4500 V; mass range, 100–1200 *m*/*z*; ion source gas 1 and gas 2 at 50 psi; curtain gas at 35 psi; interface heater temperature at 600 °C. Information-dependent acquisition (IDA) was used for MS/MS fragmentation.

To monitor instrumental stability and data quality throughout the analysis, a pooled quality control (QC) sample was prepared from equal aliquots of all samples. The QC sample was analyzed after every five experimental injections, followed by a blank solvent run. The tight clustering of QC sample data in subsequent multivariate analysis confirmed the reproducibility and stability of the system during the entire acquisition sequence.

### 2.4. Fatty Acid Methyl Ester (FAME) Preparation

Fatty acid methyl esters (FAMEs) were prepared according to a reported method [38]. Briefly, approximately 180 mg of fresh pericarp powder was mixed with 2 mL of 5% H_2_SO_4_ in methanol and 300 μL of toluene in a headspace vial. Then, 10 μL of methyl heptadecanoate solution (5 mg/mL, as internal standard) was added. The mixture was reacted at 95 °C for 1.5 h. After cooling to room temperature, 2 mL of 0.9% NaCl and 1 mL of *n*-hexane were added, followed by centrifugation at 5000 rpm for 5 min. The supernatant was collected and evaporated under nitrogen, and the residue was redissolved in 100 μL of *n*-hexane. All samples were prepared in triplicate and stored at –20 °C prior to GC analysis.

### 2.5. Gas Chromatography (GC) Analysis of Fatty Acids

FAMEs were quantified using an Agilent 7890A GC system (Santa Clara, CA, USA) equipped with a flame ionization detector (FID). Separation was achieved on a DB-FastFAME capillary column (30 m × 0.25 mm, 0.25 μm; Agilent, Santa Clara, CA, USA). The injector and detector temperatures were set at 250 °C and 260 °C, respectively. The oven temperature program was as follows: initial temperature, 80 °C, held for 0.5 min, ramped to 165 °C at 40 °C/min and held for 1 min, and then increased to 230 °C at 4 °C/min and held for 4 min. Fatty acids were identified by comparing retention times with a 37-component FAME standard mixture (Supelco FAME 37, CRM47885, Sigma-Aldrich, St. Louis, MO, USA). Relative fatty acid percentages were determined by area normalization.

### 2.6. Data Processing and Lipid Identification

Raw UPLC-MS data files were converted to Analysis Base File (.abf) format. Peak picking, alignment, and lipid identification were performed using MS-DIAL (version 4.60) with the integrated LipidBlast database. The mass tolerance was set to 0.01 Da for MS and 0.05 Da for MS/MS spectra, with an identification score cutoff of 80%. To enhance confidence, lipid identifications were further verified manually using PeakView (version 1.0) based on mass accuracy (error < 5 ppm), retention time (error < 5%), and isotopic pattern matching (difference < 10%). A quantitative method was established using MasterView (version 2.0), and detailed quantification was performed using MultiQuant (version 3.0.3). The peak area of each lipid species was normalized to the corresponding internal standard for relative quantification. Data are reported as relative concentrations.

### 2.7. Statistical Analysis

All experiments were conducted with three biological replicates, and data are presented as mean ± standard deviation (SD). The processed lipidomic data were imported into MetaboAnalyst 6.0 (http://metaboanalyst.ca (accessed on 8 March 2025) for principal component analysis (PCA), hierarchical clustering analysis (HCA), one-way analysis of variance (ANOVA), and fold-change (FC) calculation. Partial least squares-discriminant analysis (PLS-DA) and k-means clustering were performed using the Metware Cloud platform (http://cloud.metware.cn). Significantly differential lipids among three species were selected based on a variable importance in projection (VIP) score > 1.0, ANOVA *p* < 0.05, and FC > 1.5 or < 2/3. The *p*-values from the ANOVA were adjusted for multiple testing using the Benjamini–Hochberg false discovery rate (FDR) correction. Missing values were imputed with the mean of the corresponding variable.

### 2.8. Pathway Analysis

Lipid metabolic pathway analysis was conducted using MetaboAnalyst 6.0. Given the absence of a fully annotated genome for a closely related Rutaceae model, the *Arabidopsis thaliana* KEGG pathway database was selected for its superior annotation quality and comprehensive coverage of plant lipid metabolism. Pathway enrichment and topology analyses were performed using hypergeometric tests and relative betweenness centrality measures, respectively.

## 3. Results and Discussion

### 3.1. Global Lipidomic Profile of Huajiao (Zanthoxylum spp.)

**Figure 1:**
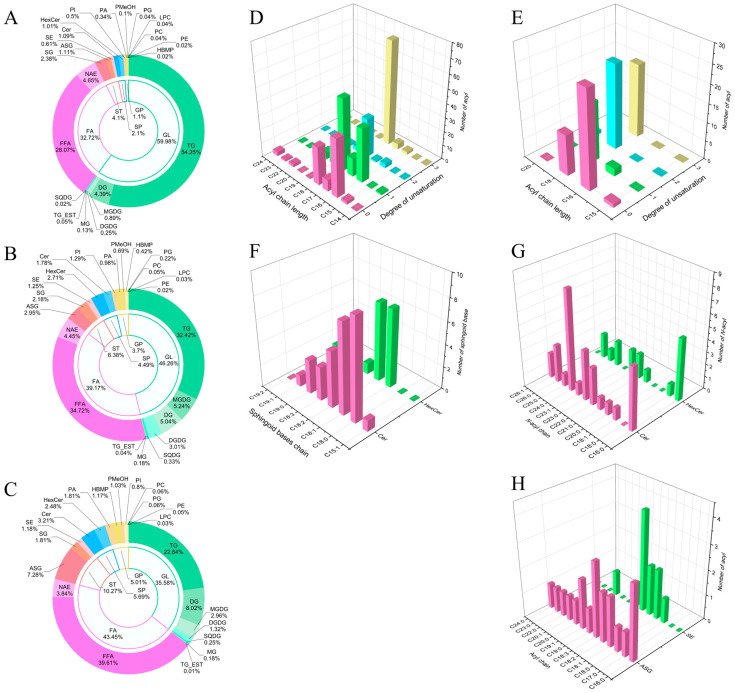
Global lipid profiles and structural characteristics in three *Zanthoxylum* species. (**A**–**C**) Relative abundance of lipid categories and subclasses in (**A**) *Z. bungeanum*, (**B**) *Z. schinifolium*, and (**C**) *Z. armatum*. (**D**,**E**) Distribution of chain length and unsaturation in fatty acyl moieties of GL (**D**) and GP (**E**). (**F**,**G**) Sphingoid base (**F**) and *N*-acyl chain (**G**) profiles in Cer and HexCer. (**H**) Acyl chain profiles in ASG and SE.

Comprehensive lipidomic analysis identified 315 lipid molecules from the fresh pericarps of three *Zanthoxylum* species: *Z. bungeanum* (RHJ), *Z. schinifolium* (GHJ), and *Z. armatum* (TJ) (Table S1; Figure S2). All concentrations reported are relative, based on internal standard normalization. These lipids were classified into five major categories: fatty acyls (FAs, 53 species), glycerolipids (GLs, 132 species), glycerophospholipids (GPs, 50 species), sphingolipids (SPs, 46 species), and sterol lipids (STs, 34 species). Further subdivision yielded 22 subclasses (Table 1). At the category level, GL (35.58–59.98%), FA (32.72–43.45%), and ST (4.10–10.27%) were the most abundant, followed by SP (2.10–5.69%) and GP (1.10–5.01%). At the subclass level, triacylglycerols (TGs, 22.84–54.25%), free fatty acids (FFAs, 28.07–39.61%), and diacylglycerols (DGs, 4.39–8.02%) predominated, with substantial amounts of *N*-acyl ethanolamines (NAEs, 3.84–4.65%), acylated steryl glycosides (ASGs, 1.11–7.28%), and monogalactosyldiacylglycerols (MGDGs, 0.89–5.24%) also present (Table 1 and Figure 1A–C). These results highlight the considerable diversity and quantitative variation in lipid composition across three *Zanthoxylum* species.

Lipid profiles exhibit both conserved and species-specific features. Due to the limited lipidomic studies on Rutaceae plants—currently restricted to *Citrus sphaerocarpa*—we also included bell pepper (*Capsicum annuum*, Solanaceae) and *Camellia oleifera* (Theaceae) for comparative analysis. A study on *C. sphaerocarpa* pericarp cultivated in Japan identified 732 lipid molecules across six categories and 45 subclasses, with GL dominating (61.41% of total lipids) and TG being the major subclass (38.54%) [39]. Huajiao contained fewer lipid molecules (315) and classes (five categories, 22 subclasses) but shared the dominance of GLs and TG. Sutliff et al. reported 106 lipids in North American bell pepper, including 36 GPs, 21 GLs, and 3 SPs, among others [23]. Bell pepper thus exhibits lower lipid diversity compared to huajiao and notably lacks FFA—a major lipid subclass in the latter. In contrast, the woody oil crop *Camellia oleifera* is characterized by an extreme predominance of TGs (91.57–93.48%), followed by DG (4.78–6.78%) and monoacylglycerol (MG, 0.59–1.05%), with other lipids occurring minimally [40]. While both huajiao and *C. oleifera* contain high levels of TG and DG, huajiao possesses substantially more FFAs. The distinct lipidomic signatures of huajiao (*Zanthoxylum* spp.) revealed by these comparisons provide a foundation for understanding Rutaceae phylogeny and functional diversity.

### 3.2. Structural Characteristics of Huajiao Lipids

Based on the LIPIDS MAPS classification system [41], five major lipid categories were identified in huajiao: FA, GL, GP, SP, and ST.

Fatty acyls (FAs), the fundamental building blocks of complex lipids, were represented by 30 FFAs and 23 NAEs. Their acyl chains ranged from C13 to C34 with 0–5 degrees of unsaturation (Table S1). C18 chains were the most prevalent, followed by C20 and C22. While FFAs contained up to three double bonds (e.g., FA 18:3 and FA 20:3), NAEs exhibited higher unsaturation, with up to five double bonds (e.g., NAE 18:5, NAE 20:5).

Long-chain fatty acyls are common structural motifs in glycerolipids (GLs) and glycerophospholipids (GPs), contributing to their molecular diversity. GLs (132 species) incorporated acyl chains ranging from C14 to C24, with C18 being the most abundant (180 chains), followed by C16 (84 chains). Monounsaturated chains predominated (120 chains), and the most common acyl groups were FA 18:3 (74 chains) and FA 18:1 (51 chains) (Figure 1D). In contrast, the 50 GPs featured acyl chains from C15 to C20, with C18 again dominant (70 chains). However, saturated chains were more prevalent in GPs (38 chains), and the predominant acyl groups were FA 16:0 (26 chains) and FA 18:2 (23 chains) (Figure 1E).

Sphingolipids (SPs), characterized by a sphingoid base and a *N*-acyl chain, included 30 ceramides (Cers) and 16 hexosylceramides (HexCers). Cer exhibited greater structural diversity in both the sphingoid base and *N*-acyl chain compared to HexCer. Sphingoid base chain lengths ranged from C15 to C19, with monounsaturated species being most common. Ceramide sphingoid bases were primarily C 18:0 and C 18:1, whereas HexCers bases were mainly C 18:1 and C 18:2 (Figure 1F). *N*-acyl chains spanned C16 to C26 and were predominantly saturated, with FA 24:0 being the most abundant in Cer, and FA 16:0 dominating in HexCer (Figure 1G).

Sterol lipids (STs) comprised 24 molecular species, categorized into acylated steryl glycosides (ASGs, 20 species), steryl glycosides (SGs, 3 species), and sterol esters (SEs, 11 species). These lipids were built on four core sterol skeletons: campesterol, sitosterol, stigmasterol, and brassicasterol. ASGs contained the first three sterol types, SEs included all four, and SGs were limited to stigmasterol. Analysis of their acyl moieties revealed that ASG structures incorporated a diverse range of acyl chains (C16–C24), which were predominantly saturated (10 out of 20 chains) and rich in C18 species (8 chains). In contrast, SEs featured fewer distinct acyl types, also predominantly C18-based (9 out of 11 chains), but exhibited a more balanced distribution between saturated and unsaturated states (Figure 1H).

### 3.3. Interspecies Variation in Lipid Composition

Lipids constitute a major class of metabolites in huajiao. Given their well-established role as key precursors to volatile aroma compounds (e.g., via lipid oxidation), their compositional profile may significantly influence flavor characteristics and could serve as a potential biochemical marker for quality evaluation. Comparative analysis revealed substantial differences between RHJ, GHJ, and TJ. The total lipid content was highest in RHJ (6965.18 ± 395.46 μg/g), followed by TJ (4388.90 ± 96.04 μg/g) and GHJ (3909.32 ± 314.31 μg/g) (Table 1). Although the three species shared the same lipid categories and molecular species, their relative abundances differed markedly. GLs were the most abundant category in RHJ (59.98%) and GHJ (46.26%), followed by FAs (32.72% and 39.17%). In contrast, TJ was dominated by FAs (43.45%), followed by GLs (35.58%) (Figure 1A–C).

#### 3.3.1. Multivariate Statistical Analysis

**Figure 2:**
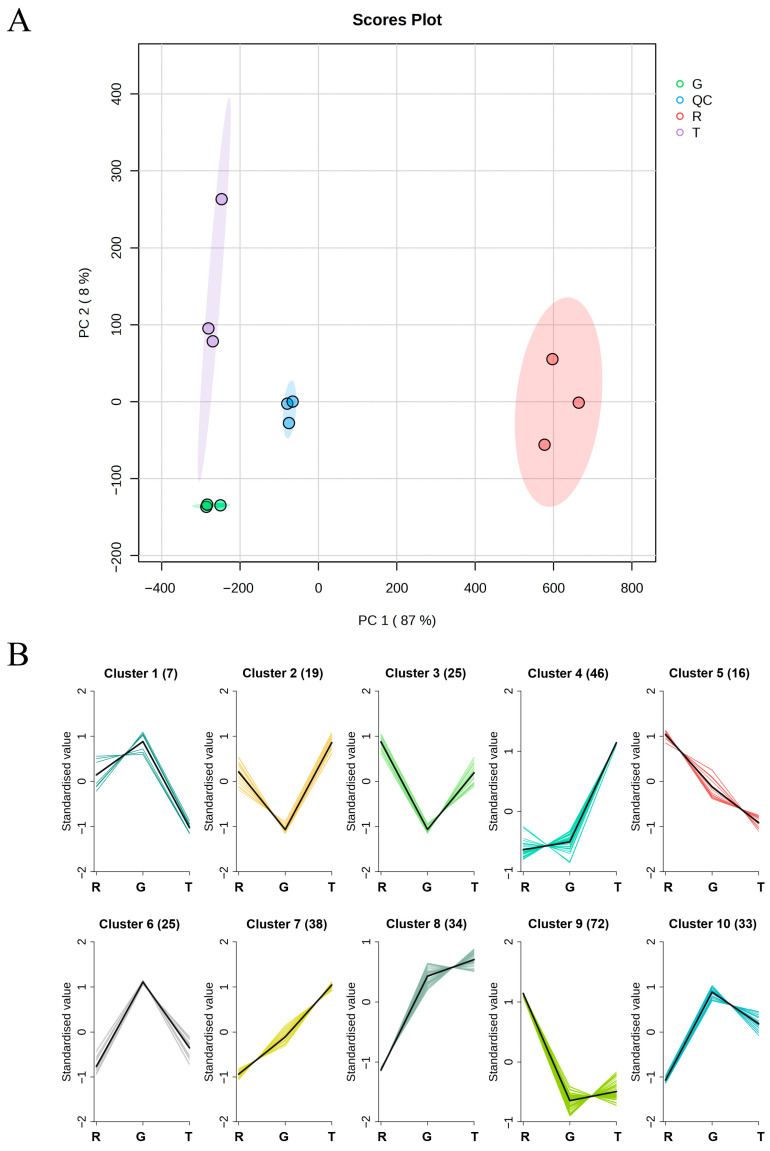
Multivariate analysis of huajiao lipidomes. (**A**) Principal component analysis (PCA) score plot revealing clear separation among three *Zanthoxylum* species. (**B**) k-means cluster analysis results, showing species-specific accumulation patterns (cluster sizes are given in parentheses). R: *Z. bungeanum*, G: *Z. schinifolium*, T: *Z. armatum*, QC: quality control.

To obtain an overview of lipid compositional differences among the three huajiao species and assess reproducibility within biological replicates, multivariate analyses were employed. Principal component analysis (PCA), an unsupervised pattern recognition technique [42], was applied to visualize global lipidomic differences. PCA clearly separated the three species, with all biological replicates clustering tightly within the 95% confidence interval, demonstrating high reproducibility. The first two principal components explained 87% (PC1) and 8% (PC2) of the total variance, effectively capturing species-specific lipidomic signatures (Figure 2A). To identify the lipid molecules responsible for this separation, the PCA loadings were examined in a biplot (Figure S3). The loading plot revealed that the segregation along the dominant PC1 axis was predominantly driven by a suite of triacylglycerols (TGs). To corroborate these findings, hierarchical cluster analysis (HCA) was applied. This unsupervised clustering method, which constructs a hierarchical tree structure to reveal data groupings [43], further confirmed the distinct separation, as evidenced by the dendrogram and heatmap, which showed clear clustering by species (Figure S4).

To explore finer-scale patterns in lipid abundance variations, k-means clustering was applied to the 315 detected lipid molecules. This unsupervised machine learning algorithm grouped lipids into 10 clusters based on their concentration trends across the three species (Table S2) [44]. Lipid category distribution within clusters showed distinct patterns: FAs and GLs were enriched in Cluster 9, GPs and SPs in Cluster 7, and STs in Cluster 4 (Table S3). Based on the abundance trends across species, the lipids were grouped into four major variation patterns: (1) Clusters 1, 6, and 10 exhibited significantly higher lipid content in GHJ than in RHJ and TJ, with no consistent difference between the latter two; (2) Clusters 2, 3, and 9 showed the opposite trend, with the lowest abundance in GHJ; (3) Clusters 4, 7, and 8 displayed a progressive increase in lipid content from RHJ to TJ, most markedly in Cluster 7; and (4) Cluster 5 showed a decreasing trend from RHJ to TJ (Figure 2B). These results highlight not only interspecies differences at the lipid subclass level but also distinct accumulation patterns among individual lipid molecules within the same subclass, indicating highly specific lipid regulatory mechanisms in each huajiao species.

#### 3.3.2. Identification of Differential Lipids

**Figure 3:**
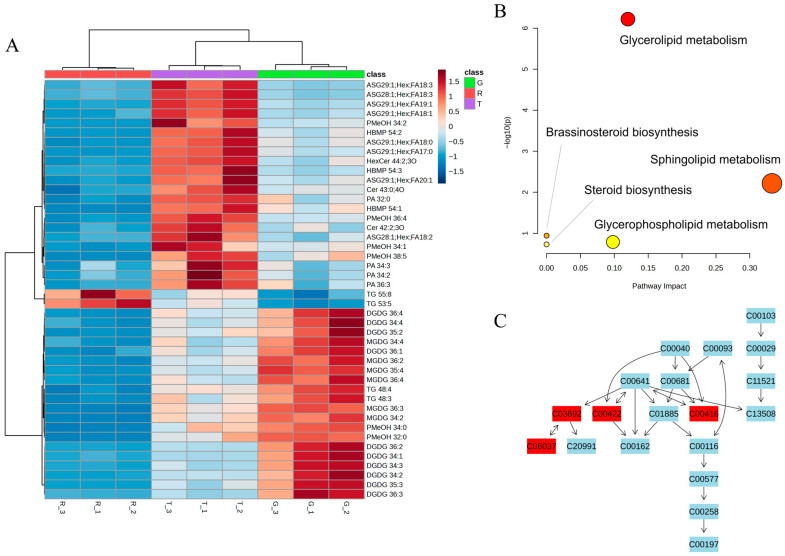
Characterization of differential lipids and their associated metabolic pathways in huajiao. (**A**) Heatmap from hierarchical cluster analysis of the 44 differential lipids across *Z. bungeanum* (R), *Z. schinifolium* (G), and *Z. armatum* (T). The complete identification details and full acyl chain compositions for all differential lipids are provided in Supplementary Table S1. (**B**) Enrichment analysis of key lipid metabolic pathways impacted by the differential lipids. (**C**) Schematic of the glycerolipid metabolism pathway; KEGG compound identifiers are shown, and differential lipids identified as key components are highlighted in red.

To identify specific lipid molecules responsible for the observed interspecies differentiation, differential analysis was performed using a combination of variable importance in projection (VIP) from partial least squares-discriminant analysis (PLS-DA), ANOVA *p*-values, and fold change (FC) criteria. A lipid was considered differential if it met the thresholds of VIP > 1.0, *p* < 0.05, and FC > 1.5 or < 2/3 in all pairwise comparisons among the three species. This rigorous approach identified 44 differential lipids, belonging to nine subclasses: TG (4 species), MGDG (6), digalactosyldiacylglycerol (DGDG, 10), phosphatidylmethanol (PMeOH, 6), hemibismonoacylglycerophosphates (HBMP, 3), phosphatidic acid (PA, 4), Cer (2), HexCer (1), and ASG (8) (Table S4). A heatmap visualization showed distinct abundance patterns: RHJ had the lowest levels for most differential lipids except TG 55:8 and TG 53:5; TJ exhibited the highest levels for half of the lipids; and GHJ showed the highest abundance for 20 lipids (Figure 3A). While certain subclasses (e.g., DGDG, MGDG, PA, HBMP, Cer, and ASG) showed consistent variation trends across species, others (e.g., PMeOH and TG) exhibited divergent patterns, underscoring the complexity of lipid compositional differences.

To elucidate the metabolic pathways associated with the differential lipids identified in the three huajiao species, the 44 differential lipids were mapped to the Kyoto Encyclopedia of Genes and Genomes (KEGG) for ID retrieval. Pathway enrichment analysis using MetaboAnalyst 6.0 revealed five significantly enriched metabolic pathways (Figure 3B). Glycerolipid metabolism was the most significantly affected (−log_10_(*p*) = 6.22, impact = 0.12), followed by sphingolipid metabolism (−log_10_(*p*) = 2.22, impact = 0.33). Key differential lipids mapped to the glycerolipid metabolism pathway included PA (C00416), TG (C00422), MGDG (C03692), and DGDG (C06037) (Figure 3C). The schematic of this pathway illustrates that PA can be hydrolyzed by lipid phosphate phosphatase (LPP) to generate DG, which in turn can be re-esterified to PA by diacylglycerol kinase (DGK) or acylated to TG by diacylglycerol acyltransferase (DGAT). Alternatively, DG can be glycosylated to MGDG by MGDG synthase, and subsequently to DGDG by DGDG synthase. A comparative analysis of the content of these key lipids across the three species revealed distinct metabolic divergences: MGDG, DGDG, and TG were most abundant in GHJ and least abundant in RHJ, whereas PA reached its highest level in TJ and its lowest in RHJ. These differential accumulation patterns underscore significant differences in the flux of glycerolipid metabolism among the three huajiao species. The prominence of the glycerolipid metabolism pathway suggests its potential role in shaping species-specific traits, including adaptation to distinct growing environments and the formation of flavor precursors. Consequently, the identified lipids provide strong candidates for future investigations into the biochemical basis of huajiao’s distinct ecology, flavor, and bioactivity. Directly linking these lipids to sensory properties, however, would require integrated volatilomic and sensory studies.

#### 3.3.3. Variations in Glycerolipids

**Figure 4:**
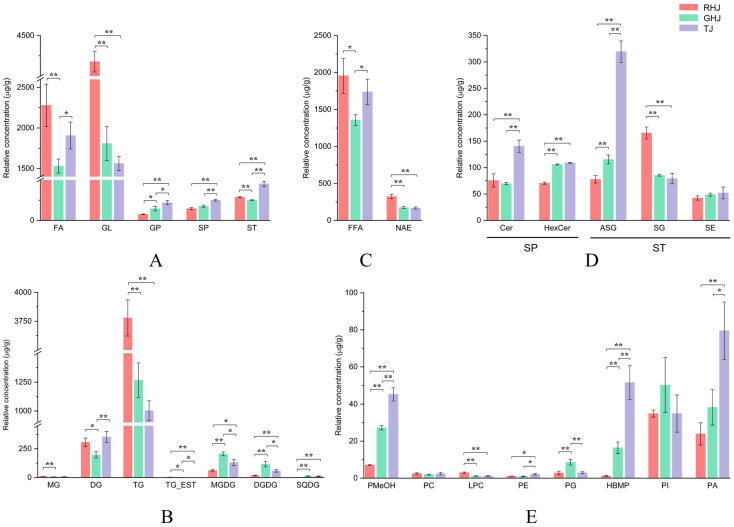
Comparative abundance of lipid categories and subclasses across three *Zanthoxylum* species. Stacked bar charts show the relative concentration of (**A**) total lipid categories and relative abundance of subclasses within (**B**) GL, (**C**) FA, (**D**) SP and ST, and (**E**) GP. Values are mean ± SD (*n* = 3). * *p* < 0.05, ** *p* < 0.01 indicate significant differences. RHJ: *Z. bungeanum*, GHJ: *Z. schinifolium*, TJ: *Z. armatum*.

Glycerolipids (GLs), essential for energy storage and as metabolic intermediates in plants [45], were the most abundant lipid category in all three species (Figure 4A). The 132 GL molecules were classified into seven subclasses: MG, DG, TG, triacylglycerol estolide (TG_EST), MGDG, DGDG, and sulfoquinovosyl diacylglycerol (SQDG) (Figure 4B). TG was the predominant subclass, especially in RHJ (90.45% of total GL), followed by GHJ (70.09%) and TJ (64.20%). DG was the second most abundant subclass in RHJ (7.32%) and TJ (22.54%), while MGDG ranked second in GHJ (11.33%). Although the subclasses were consistent across species, their molecular distribution differed markedly. RHJ’s GL profile was heavily dominated by TG, whereas GHJ and TJ exhibited more balanced distribution.

In plants, TG acts as a major energy storage lipid, accumulating abundantly in seeds and fruits to support germination and development [46]. The molecular composition of dominant TGs also varied. In RHJ, the top four TGs—TG (16:0/16:1/18:1), TG (16:1/16:1/18:1), TG (16:0/18:1/18:2), and TG (16:1/18:1/18:2)—collectively accounted for 44.76% of the total TG. In GHJ and TJ, however, TG (16:1/16:1/18:1) was the dominant species, accompanied significantly by TG (16:1/16:1/16:1) and TG (16:0/16:1/16:1); these three TGs constituted 54.46% and 44.64% of the total TG in GHJ and TJ, respectively. Notably, the most abundant TGs in RHJ were characterized by fatty acyl chains with relatively high average chain lengths and degrees of unsaturation compared to those in GHJ and TJ. Galactolipids MGDG and DGDG, though less abundant, are critical structural components of chloroplast membranes and are essential for photosynthesis [47].

#### 3.3.4. Variations in Fatty Acids

Huajiao is rich in fatty acyls (FAs), with 53 FAs identified across the three species, including 30 free fatty acids (FFAs) and 23 *N*-acyl ethanolamines (NAE) (Figure 4C). Notably, no FAs were among the 44 differential lipids, indicating relatively stable FA profiles across species. FFAs, derived mainly from storage lipid hydrolysis, serve as energy substrates and metabolic precursors [48]. In all three species, C18 fatty acids were the predominant FFA constituents (57.36–62.64% of total FFAs), including FA 18:0 (stearic acid), 18:1 (oleic acid), 18:2 (linoleic acid), and FA 18:3 (α-linolenic acid, ALA), with ALA being the most abundant species. As an essential polyunsaturated fatty acid, ALA is partially converted in the body to eicosapentaenoic acid (EPA) and docosahexaenoic acid (DHA), contributing to cardiovascular health and other physiological benefits [49]. NAEs, which are signaling lipids generated from *N*-acyl-ethanolamine phospholipid (NAPE) hydrolysis by NAPE-phospholipase D (NAPE-PLD)-accounted for 8.83–14.21% of total FAs. The major NAEs included NAEs 16:2, 20:2, and 15:4. NAEs are known to regulate plant immunity and programmed cell death and have been reported to possess anti-inflammatory, immunomodulatory, and neuroprotective properties in mammalian systems [50,51,52].

#### 3.3.5. Variations in Sterol Lipids

Sterol lipids (STs) are critical structural components of cell membranes. A total of 34 STs were identified from huajiao, categorized into three subclasses: ASG (20 species), SG (3 species), and SE (11 species) (Figure 4D). These lipids are built on four core sterols: campesterol, sitosterol, stigmasterol, and brassicasterol. Phytosterols play vital roles in membrane integrity, signaling, stress tolerance, and plant defense [53] and also exhibit bioactive properties in humans, such as cholesterol metabolism regulation, anti-inflammatory effects, and immunomodulation [54]. The distribution of ST subclasses varied notably among species: SG predominated in RHJ (57.92% of total STs), whereas ASG was dominant in GHJ (46.26%) and TJ (70.87%). At the molecular level, SG 29:1;O;Hex was most abundant in RHJ (48.95%) and GHJ (30.20%), while ASG 29:1;O;Hex;FA 16:0 and ASG 29:1;O;Hex;FA 18:3 were predominant in TJ (collectively 44.93%). As sterol conjugates, SG and ASG function as membrane stabilizers, sterol storage forms, transporters, and signaling molecules in plants [55].

#### 3.3.6. Variations in Sphingolipids and Glycerophospholipids

Sphingolipids (SPs), characterized by a sphingoid-base backbone, are essential components of plant and animal membranes [56]. A total of 46 SPs were identified in huajiao, including 30 Cers and 16 HexCers. Cer was the major SP subclass in RHJ (51.77%) and TJ (56.34%), whereas HexCer dominated in GHJ (60.35%) (Figure 4D). Ceramides are key signaling molecules that regulate critical cellular processes, including cell differentiation, proliferation, and apoptosis [57]. In this study, the ceramides identified in huajiao were categorized into six distinct types based on structural variations in their sphingoid base and *N*-acyl modifications (Table S1). Cer_AP (alpha-hydroxy fatty acid–phytospingosine) was predominant in RHJ (60.02%) and GHJ (51.67%), while Cer_AS (alpha-hydroxy fatty acid–sphingosine) was most abundant in TJ (43.31%), differing primarily in their sphingoid bases (phytosphingosine vs. sphingosine).

Glycerophospholipids (GPs), vital for membrane structure and signaling [58], were the least abundant lipid category overall. Eight GP subclasses were identified: PMeOH, phosphatidylcholine (PC), lysophosphatidylcholine (LPC), phosphatidylethanolamine (PE), phosphatidylglycerol (PG), HBMP, phosphatidylinositol (PI), and PA (Figure 4E). Despite low abundance, PIs were prominent in RHJ (45.61% of total GPs) and GHJ (34.73%), whereas PA dominated in TJ (36.15%), suggesting species-specific differences in membrane lipid composition and signaling capacity.

### 3.4. Fatty Acid Composition Analysis

Gas chromatography (GC) analysis demonstrated significant differences in oil content among fresh pericarps. RHJ (*Z. bungeanum*) and TJ (*Z. armatum*) had relatively high oil contents (6.04 ± 0.31 mg/g and 6.69 ± 0.02 mg/g, respectively), while GHJ (*Z. schinifolium*) contained less (4.52 ± 0.17 mg/g) (Table S5). These findings align with previous reports indicating substantial variations in pericarp oil content among different *Zanthoxylum* species. For instance, Zhao et al. observed that dried pericarps of *Z. bungeanum* contained 341.79 mg/g of oil, exceeding the 290.59 mg/g found in dried *Z. armatum* pericarps [18]. This comparison also highlights that dried samples generally possess higher oil content than fresh pericarps, likely due to moisture loss during dehydration.

A total of 23 fatty acids were identified and quantified, including 12 saturated fatty acids (SFAs), 6 monounsaturated fatty acids (MUFAs), and 5 polyunsaturated fatty acids (PUFAs) (Table 2). In all three species, SFAs constituted the dominant category (62.32–64.20% of total fatty acids), followed by PUFAs (19.38–22.07%) and MUFAs (15.25–16.60%) (Figure S5). Although the types of fatty acids were consistent across species, their relative abundances differed significantly. PCA clearly separated the species based on their fatty acid profiles, with the first two principal components explaining 81.9% and 12.3% of the variance, respectively (Figure S6).

Categorization by chain length showed that SFAs included three medium-chain (C6–C12) and nine long-chain (>C12) fatty acids [59]. Medium-chain SFAs represented 45.80–49.22% of total fatty acids, while long-chain SFAs accounted for 14.80–16.52%. The predominant individual fatty acids varied across species: RHJ was characterized by high C8:0 (caprylic acid, 31.99%), C16:0 (palmitic acid, 11.41%), and C18:3n3 (α-linolenic acid, 10.67%); GHJ by C8:0 (22.32%), C6:0 (caproic acid, 18.24%), and C16:0 (10.45%); and TJ by C6:0 (20.07%), C8:0 (19.75%), and C16:1 (palmitoleic acid, 9.98%). Overall, medium-chain fatty acids (C6:0, C8:0) dominated the profiles, with C18:3n3 being the major unsaturated fatty acid (9.49–10.67%) (Table 2). Comparing these results with the existing literature reveals notable discrepancies. Zhao et al. identified 12 fatty acids in dried pericarps of *Z. bungeanum* and *Z. armatum*, with PUFAs being predominant in *Z. bungeanum* (51.27%) and MUFAs most abundant in *Z. armatum* (39.74%) [18]. Another study on dried *Z. bungeanum* reported only nine fatty acids, with SFAs as the major class and C16:0, C18:3, and C18:1 as the most abundant [60]. These marked differences in the number, predominant classes, and specific profile of fatty acids between our study (on fresh pericarps) and prior reports (on dried materials) can be primarily attributed to the sample state. The drying process may not only concentrate non-volatile lipids (explaining the generally higher oil content in dried samples) but also drive the loss of volatile medium-chain fatty acids (MCFAs) and promote oxidative transformations of unsaturated fatty acids. Therefore, the high abundance of MCFAs (e.g., C8:0) observed here likely represents a genuine characteristic of fresh huajiao pericarps, which may have been underestimated in previous studies on dried products. Additionally, the specific cultivars and growth conditions investigated may also contribute to this distinct MCFA-rich profile.

The polyunsaturated fatty acids n-3 (omega-3) and n-6 (omega-6) play crucial roles in human health [61,62,63], but their balance is critical. The ideal n-6/n-3 ratio is considered to be between 1:1 and 4:1 [64]. Modern diets are often characterized by an excessively high n-6/n-3 ratio, which is linked to an increased risk of chronic diseases [65]. In stark contrast, the n-6/n-3 ratio in the three huajiao species ranged from a well-balanced 1.04 to 1.12 (Table 2). This well-balanced ratio positions huajiao as a promising functional ingredient for nutraceuticals and health-promoting foods specifically designed to counteract the prevalent dietary deficiency of omega-3 fatty acids and mitigate associated health risks.

## 4. Conclusions

This study ventures beyond the traditional focus on volatiles by presenting the first comprehensive comparative lipidomic analysis of three economically significant huajiao species (*Z. bungeanum*, *Z. schinifolium*, and *Z. armatum*) using UPLC-Q-TOF-MS/MS and GC-FID. We identified and characterized 315 lipid molecules, revealing significant interspecies variations in lipid categories and subclasses. Multivariate statistics confirmed distinct lipidomic signatures, and pathway analysis highlighted glycerolipid metabolism as a key differentiator. Fatty acid profiling further revealed a balanced n-6/n-3 PUFA ratio. While acknowledging limitations in relative quantification, genetic-environmental discrimination, and the need to validate the proposed links to flavor profiles, this work lays a foundational dataset for the development of value-added applications from *Zanthoxylum* species. These findings highlight the potential of *Zanthoxylum* lipids as discriminative markers for food authentication, particularly for identification between closely related or morphologically similar varieties, and as novel sources of balanced lipids for developing functional foods with distinctive flavor profiles and enhanced nutritional value.

## Figures and Tables

**Table 1:** Lipid content (relative concentration, μg/g) by category and subclass in three *Zanthoxylum* species.

Lipid Categories	Lipid Subclasses	Number of Identified Lipid Molecules	RHJ (*Z. bungeanum*)		GHJ (*Z. schinifolium*)		TJ (*Z. armatum*)	
			Content (μg/g)	Relative Abundance (%)	Content (μg/g)	Relative Abundance (%)	Content (μg/g)	Relative Abundance (%)
Fatty acyls (FAs)	FFA	30	1960 ± 240	28.07	1360 ± 70	34.72	1740 ± 170	39.61
	NAE	23	233 ± 30	4.65	174 ± 14	4.45	168 ± 14	3.84
	**Sum**	**53**	**2280 ± 260**	**32.72**	**1530 ± 90**	**39.17**	**1910 ± 170**	**43.45**
Glycerolipids (GLs)	MG	2	9.0 ± 0.5	0.13	7.1 ± 0.4	0.18	8.0 ± 0.5	0.18
	DG	27	306 ± 37	4.39	197 ± 27	5.04	352 ± 47	8.02
	TG	61	3780 ± 160	54.25	1270 ± 150	32.42	1000 ± 90	22.84
	TG_EST	4	3.0 ± 0.6	0.05	1.6 ± 0.4	0.04	0.64 ± 0.14	0.01
	MGDG	18	61.8 ± 5.6	0.89	205 ± 16	5.24	130 ± 26	2.96
	DGDG	13	17.6 ± 2.8	0.25	117 ± 24	3.01	58 ± 11	1.32
	SQDG	7	1.6 ± 0.3	0.02	12.8 ± 2.4	0.33	10.8 ± 2.6	0.25
	**Sum**	**132**	**4180 ± 130**	**59.98**	**1810 ± 210**	**46.26**	**1560 ± 90**	**35.58**
Glycerophospholipids (GPs)	PMeOH	10	7.11 ± 0.25	0.10	27.2 ± 1.2	0.69	45.3 ± 3.6	1.03
	PC	6	2.5 ± 0.4	0.04	1.9 ± 0.2	0.05	2.5 ± 0.7	0.06
	LPC	4	3.0 ± 0.3	0.04	1.17 ± 0.10	0.03	1.16 ± 0.22	0.03
	PE	5	1.11 ± 0.09	0.02	0.93 ± 0.17	0.02	2.14 ± 0.46	0.05
	PG	9	2.8 ± 0.9	0.04	8.6 ± 1.5	0.22	3.0 ± 0.5	0.06
	HBMP	4	1.2 ± 0.3	0.02	16.4 ± 3.1	0.42	51.6 ± 9.2	1.17
	PI	3	34.9 ± 1.9	0.50	50 ± 15	1.29	35 ± 10	0.80
	PA	9	24 ± 6	0.34	38 ± 10	0.98	80 ± 15	1.81
	**Sum**	**50**	**76 ± 3**	**1.10**	**145 ± 27**	**3.70**	**220 ± 26**	**5.01**
Sphingolipids (SPs)	Cer	30	76 ± 12	1.09	69.6 ± 2.0	1.78	141 ± 11	3.21
	HexCer	16	70.6 ± 2.0	1.01	106 ± 13	2.71	109.0 ± 0.9	2.48
	**Sum**	**46**	**146 ± 14**	**2.10**	**175 ± 13**	**4.49**	**250 ± 11**	**5.69**
Sterol lipids (STs)	ASG	20	78 ± 7	1.11	115 ± 8	2.95	320 ± 20	7.28
	SG	3	166 ± 11	2.38	85.2 ± 1.4	2.18	79 ± 10	1.81
	SE	11	42 ± 4	0.61	49.0 ± 2.8	1.25	52 ± 11	1.18
	**Sum**	**34**	**286 ± 6**	**4.10**	**250 ± 6**	**6.38**	**451 ± 33**	**10.27**
**Total**	**22**	**315**	**6970 ± 400**	**100.00**	**3910 ± 310**	**100.00**	**4390 ± 100**	**100.00**

Values are presented as mean ± SD (*n* = 3); % is the percentage of a certain subclass of lipids to the total lipids.

**Table 2:** Relative fatty acid composition of three *Zanthoxylum* species (%).

Fatty Acids	Red Huajiao (*Z. bungeanum*)	Green Huajiao (*Z. schinifolium*)	Tengjiao (*Z. armatum*)
C6:0	10.03	18.24	20.07
C8:0	31.99	22.32	19.75
C11:0	3.78	7.67	9.40
C13:0	0.97	1.61	1.81
C14:0	0.42	0.08	0.42
C15:0	0.17	0.41	0.39
C16:0	11.41	10.45	9.30
C18:0	1.19	1.00	0.78
C20:0	0.14	0.48	0.97
C22:0	0.12	0.95	0.55
C23:0	0.19	0.40	0.23
C24:0	1.91	0.59	0.35
**ΣSFA**	**62.32**	**64.20**	**64.02**
C14:1	0.17	0.17	0.14
C15:1	0.13	0.38	0.15
C16:1	4.01	8.04	9.98
C17:1	0.87	0.68	0.67
C18:1n9t	8.22	2.98	2.89
C18:1n9c	2.21	3.00	2.77
**ΣMUFA**	**15.61**	**15.25**	**16.60**
C18:2n6t	0.48	0.03	0.07
C18:2n6c	9.02	7.84	6.66
C18:3n6	1.81	2.92	3.10
C18:3n3	10.67	9.62	9.49
C20:2	0.09	0.14	0.06
**ΣPUFA**	**22.07**	**20.55**	**19.38**
Σn-3PUFA	10.67	9.62	9.49
Σn-6PUFA	11.31	10.79	9.83
n-6/n-3	1.06	1.12	1.04

SFA: saturated fatty acid, MUFA: monounsaturated fatty acid, PUFA: polyunsaturated fatty acid, n-3: omega-3 fatty acids; n-6: omega-6 fatty acids.

## Data Availability

The original contributions presented in this study are included in the article/Supplementary Material. Further inquiries can be directed to the corresponding author.

## Supplementary Materials

The following supporting information can be downloaded at https://www.mdpi.com/article/10.3390/foods15020372/s1, Figure S1: Morphological features of three *Zanthoxylum* species; Figure S2: Total ion chromatogram (TIC) of lipid profiles in the fresh pericarps of three *Zanthoxylum* species; Figure S3: Principal component analysis (PCA) biplot of lipidomic profiles; Figure S4; Hierarchical clustering analysis (HCA) heatmap of the lipid profiles from three *Zanthoxylum* species; Figure S5: Fatty acids composition of three *Zanthoxylum* species; Figure S6: Principal component analysis (PCA) score plot of all fatty acid molecules across three *Zanthoxylum* species; Table S1: The total lipid molecules identified from three *Zanthoxylum* species; Table S2: Results of k-means cluster analysis; Table S3: Numbers of lipid molecules of FA, GL, GP, SP and ST in k-means cluster analysis; Table S4: The differential lipid molecules screened out; Table S5: The total fatty acids identified from three *Zanthoxylum* species using GC-FID.

## Abbreviations

The following abbreviations are used in this manuscript:

UPLC	Ultra-performance liquid chromatography
Q-TOF	Quadrupole time-of-flight
MS	Mass spectrometry
GC	Gas chromatography
RHJ	Red huajiao (*Zanthoxylum bungeanum*)
GHJ	Green huajiao (*Zanthoxylum schinifolium*)
TJ	Tengjiao (*Zanthoxylum armatum*)
ACN	Acetonitrile
IPA	Isopropanol
FA	Fatty acyl
GL	Glycerolipid
GP	Glycerophospholipid
SP	Sphingolipid
ST	Sterol lipid
FFA	Free fatty acid
NAE	*N*-acyl ethanolamine
MG	Monoacylglycerol
DG	Diacylglycerol
TG	Triacylglycerol
TG_EST	Triacylglycerol estolide
MGDG	Monogalactosyldiacylglycerol
DGDG	Digalactosyldiacylglycerol
SQDG	Sulfoquinovosyl diacylglycerol
PMeOH	Phosphatidylmethanol
PC	Phosphatidylcholine
LPC	Lysophosphatidylcholine
PE	Phosphatidylethanolamine
PG	Phosphatidylglycerol
HBMP	Hemibismonoacylglycerophosphate
PI	Phosphatidylinositol
PA	Phosphatidic acid
Cer	Ceramide
HexCer	Hexosylceramide
ASG	Acylated steryl glycoside
SG	Steryl glycoside
SE	Sterol ester
SFA	Saturated fatty acid
MUFA	Monounsaturated fatty acid
PUFA	Polyunsaturated fatty acid
FAME	Fatty acid methyl ester
PCA	Principal component analysis
HCA	Hierarchical clustering analysis
