# Supplementary material for: Comparative Lipidomics Unveils Species-Specific Lipid Signatures in Three Zanthoxylum Species
Source: Foods. 2026 Jan 20;15(2):372. doi: 10.3390/foods15020372 (PMC12841136; doi:10.3390/foods15020372)
Supplement: Supplementary file 1 [file foods-15-00372-s001.zip › Table S3, Figure S1-S6.pdf]

Table S3. Numbers of lipid molecules of FA, GL, GP, SP and ST in k-means cluster analysis.

Cluster No.	FA	GL	GP	SP	ST	Total
Cluster 1	1	2	2	1	1	7
Cluster 2	5	9	1	3	1	19
Cluster 3	6	13	2	3	1	25
Cluster 4	3	5	11	9	**18**	46
Cluster 5	4	10	1	1	0	16
Cluster 6	0	16	7	2	0	25
Cluster 7	5	2	**15**	**11**	5	38
Cluster 8	8	10	5	7	4	34
Cluster 9	**16**	**44**	4	4	4	72
Cluster 10	5	21	2	5	0	33

FA: fatty acyls, GL: glycerolipids, GP: glycerophospholipids, SP: sphingolipids, ST: sterol lipids.

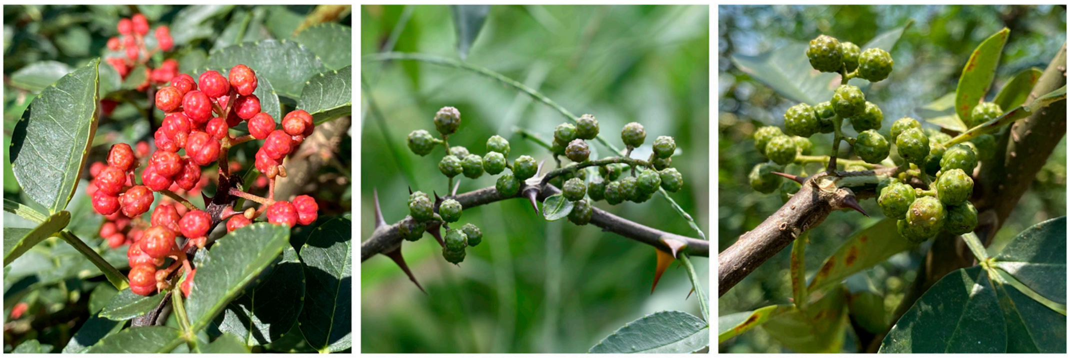

RHJ (*Z. bungeanum*) GHJ (*Z. schinifolium*) TJ (*Z. armatum*)

Figure S1. Morphological features of three *Zanthoxylum* species.

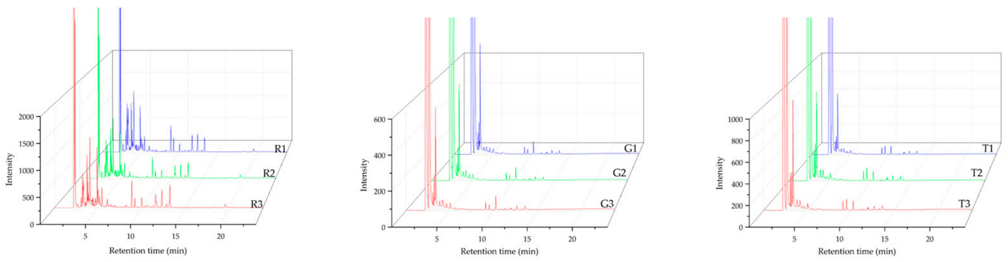

A B C

Figure S2. Total ion chromatogram (TIC) of lipid profiles in the fresh pericarps of three *Zanthoxylum* species: (**A**) red huajiao (RHJ, *Z. bungeanum*), (**B**) green huajiao (GHJ, *Z. schinifolium*) and (**C**) tengjiao (TJ, *Z. armatum*). R1, R2 and R3: three biological replicates of RHJ; G1, G2 and G3: three biological replicates of GHJ; T1, T2 and T3: three biological replicates of TJ.

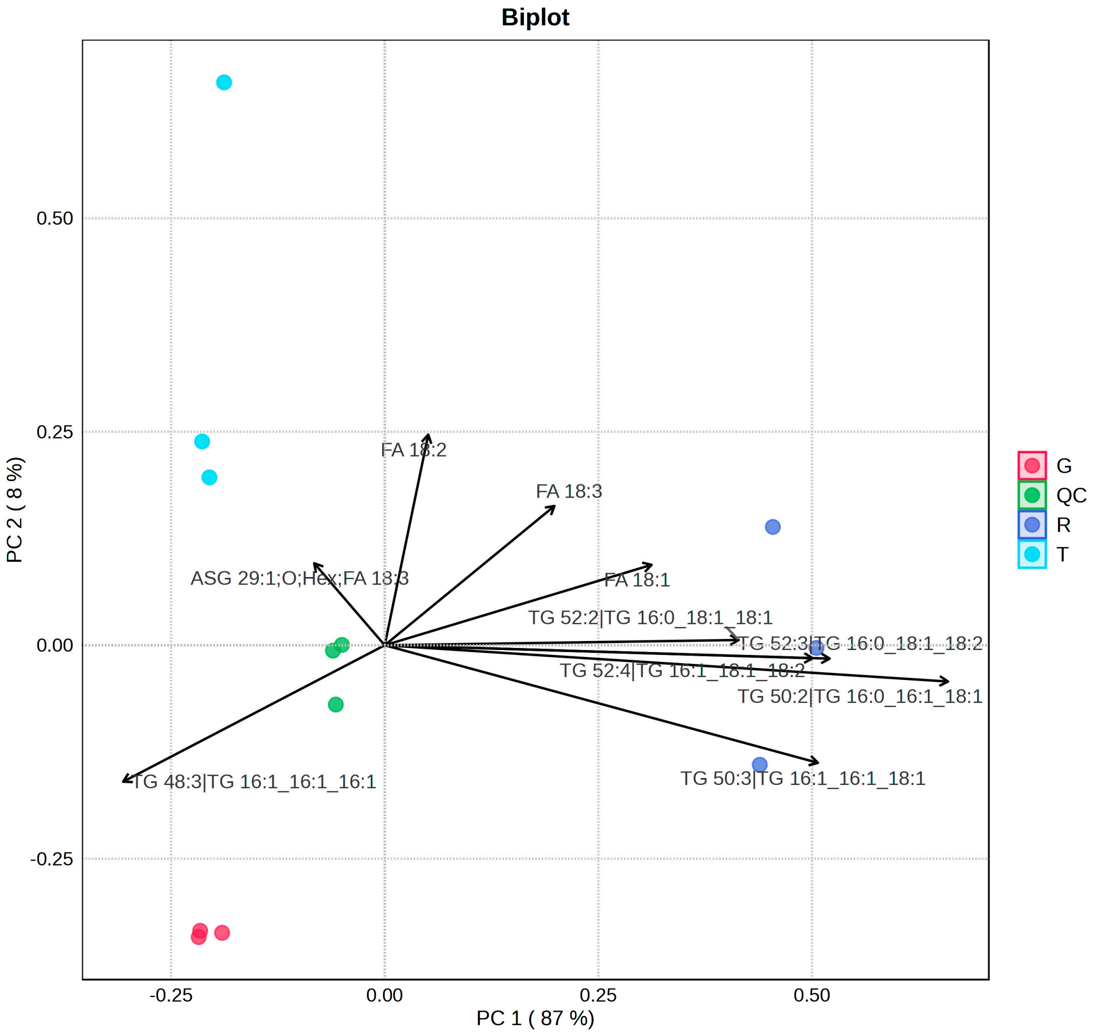

Figure S3. Principal component analysis (PCA) biplot of lipidomic profiles. The plot displays both sample scores (points) and variable loadings (vectors/labels). Score points represent individual samples, and loading vectors (labeles) indicate the direction and influence of individual lipid molecules on the principal components; only the top contributing lipids are labeled for clarity. R: *Z. bungeanum*, G: *Z. schinifolium*, T: *Z. armatum*, QC: quality control.

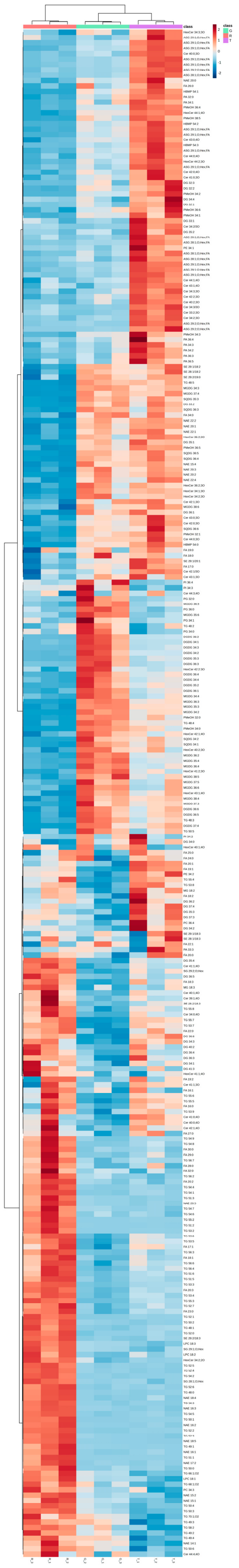

Figure S4. Hierarchical clustering analysis (HCA) heatmap of the lipid profiles from three *Zanthoxylum* species. Rows represent individual lipid molecules, and the color intensity in each cell corresponds to their relative abundance. R: *Z. bungeanum*, G: *Z. schinifolium*, T: *Z. armatum*.

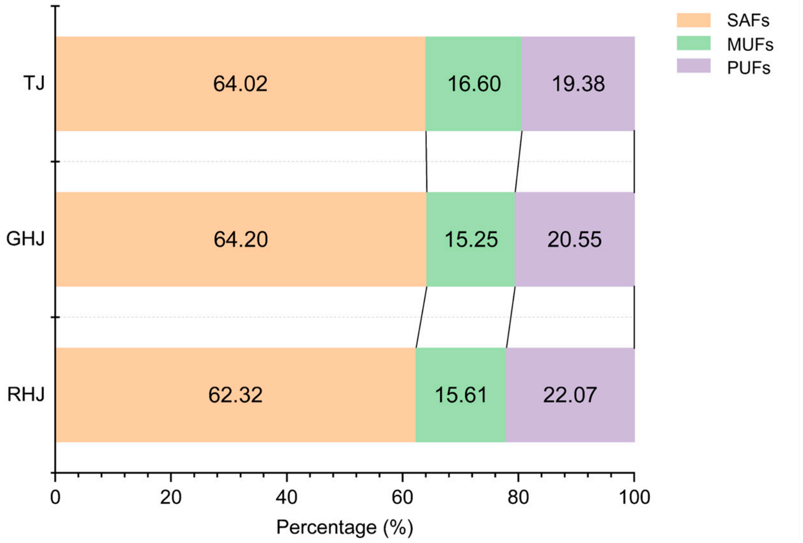

Figure S5. Fatty acids composition of three *Zanthoxylum* species. RHJ: *Z. bungeanum*, GHJ: *Z. schinifolium*, TJ: *Z. armatum*, SAFs: saturated fatty acids, MUFs: monounsaturated fatty acids, PUFs: polyunsaturated fatty acids.

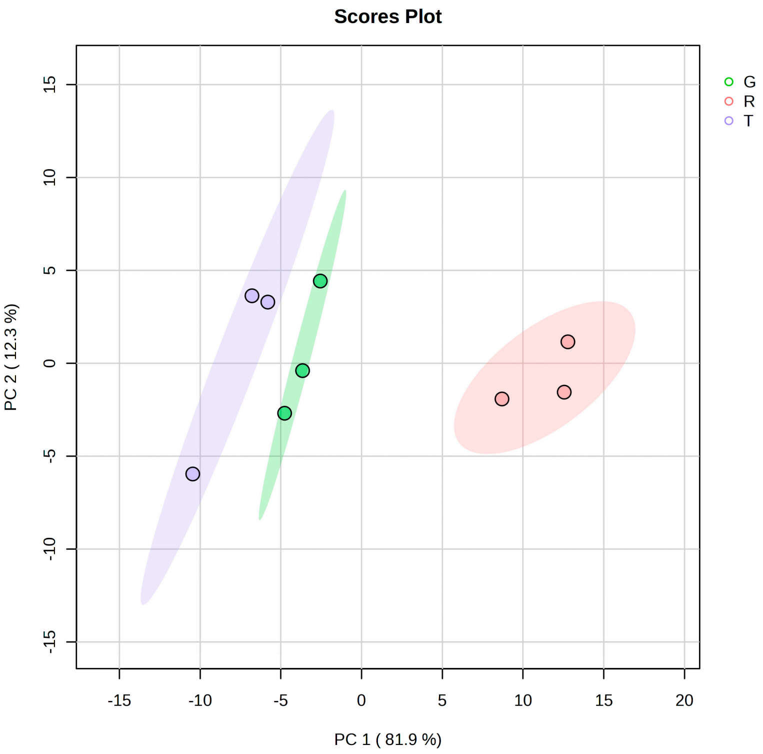

Figure S6. Principal component analysis (PCA) score plot of all fatty acid molecules across three *Zanthoxylum* species. R: *Z. bungeanum*, G: *Z. schinifolium*, T: *Z. armatum*.
